# Genome Sequence of *Lactobacillus plantarum* 19L3, a Strain Proposed as a Starter Culture for Slovenská Bryndza Ovine Cheese

**DOI:** 10.1128/genomeA.00292-14

**Published:** 2014-04-24

**Authors:** Giuseppe D'Auria, Mária Džunková, Andrés Moya, Martin Tomáška, Miroslav Kološta, Vladimir Kmet

**Affiliations:** a Área de Genómica y Salud, Fundación para el Fomento de la Investigación Sanitaria y Biomédica de la Comunidad Valenciana (FISABIO-Salud Pública), Valencia, Spain, and Instituto Cavanilles de Biodiversidad y Biología Evolutiva, Universitat de València, Paterna, Valencia, Spain; b CIBER en Epidemiología y Salud Pública (CIBEResp), Madrid, Spain; c Dairy Research Institute, Žilina, Slovakia; d Institute of Animal Physiology, Slovak Academy of Sciences, Košice, Slovakia

## Abstract

The genome sequence of *Lactobacillus plantarum* isolated from ovine cheese is presented here. This bacterium is proposed as a starter strain, named 19L3, for Slovenská bryndza cheese, a traditional Slovak cheese fulfilling European Food Safety Authority (EFSA) requirements.

## GENOME ANNOUNCEMENT

*Lactobacillus plantarum* 19L3 was isolated from sheep's milk cheese in Slovakia during a search for novel strains to be used as starter cultures to produce the traditional Slovak ovine cheese Slovenská bryndza (1) from pasteurized sheep milk. Cottage cheese known as “lump cheese” is a traditional sheep's milk dairy product made in Slovakia. This denomination is listed in the European Union registry of traditional specialties guaranteed (TSG). It is made from unpasteurized sheep's milk without any additional dairy starters, and only the natural microbiota is involved in short-time ripening (several days at different temperatures). The final product is semihard, with a typical lactic acid smell and taste reminiscent of sheep's milk. It can be consumed fresh or smoked, or processed to bryndza, another European Union-protected Slovak dairy specialty. Slovak bryndza is crushed and salted lump cheese made with sheep's milk. New hygiene regulations necessitate changes in the production process. The sheep's milk must be first pasteurized, and then cheese-forming starter cultures of *Lactobacillus*, *Lactococcus*, *Enterococcus*, and yeast (*Geotrichum bryndzae*) are added for cheese maturation (2, 3). Therefore, there is a need to find novel starter culture strains fulfilling European Food Safety Authority (EFSA) requirements.

Laboratory evaluation showed that *L. plantarum* 19L3 does not produce biogenic amines, which is one of the EFSA requirements. Moreover, it does not produce CO_2_, which causes swelling, one of the criteria required for consumption by children, nor does it produce beta-glucuronidases, which are a potential risk factor for large intestine cancer (4). Its biochemical features include good proteolytic activity and tolerance to NaCl (up to 4%). Moreover, it is susceptible to antibiotics, according to the ISO 10932 standard (5, 6). The objective of performing whole-genome sequencing was to investigate the presence of antibiotic resistance or virulence factors, which could not be evaluated by laboratory testing.

The genome of *L. plantarum* was sequenced using a Roche 454 GS FLX Titanium sequencer platform. All 47,291,746 obtained reads (with mode lengths of 479 bp) were assembled *de novo* using the MIRA 3 assembler (7). The assembly resulted in 241 contigs (203 contigs of >1,000 bp). The draft genome assembly included in total 3,305,502 bp with an average coverage of 13.06× and a GC content of 44%. Alignment of the resulting contigs against the nr database by NCBI-blast (8) using the Megablast algorithm confirmed similarity with *Lactobacillus plantarum* (accession no. 254044096). Open reading frames (ORFs) were identified by Glimmer 3 (9) and annotated by InterProScan Sequence Search (10) based on Pfam hidden Markov models (11).

### Nucleotide sequence accession number.

The genome sequence has been deposited in GenBank under the accession number AWTS00000000.
